# The Genetic Architecture of Maize Stalk Strength

**DOI:** 10.1371/journal.pone.0067066

**Published:** 2013-06-20

**Authors:** Jason A. Peiffer, Sherry A. Flint-Garcia, Natalia De Leon, Michael D. McMullen, Shawn M. Kaeppler, Edward S. Buckler

**Affiliations:** 1 Department of Plant Breeding and Genetics, Cornell University, Ithaca, New York, United States of America; 2 Plant Genetics Research Unit, USDA-Agricultural Research Service and Division of Plant Sciences, University of Missouri, Columbia, Missouri, United States of America; 3 Department of Agronomy, University of Wisconsin, Madison, Wisconsin, United States of America; 4 United States Department of Agriculture - Agricultural Research Service, Robert W. Holley Center for Agriculture and Health, Ithaca, New York, United States of America; University of Nottingham, United Kingdom

## Abstract

Stalk strength is an important trait in maize (Zea mays L.). Strong stalks reduce lodging and maximize harvestable yield. Studies show rind penetrometer resistance (RPR), or the force required to pierce a stalk rind with a spike, is a valid approximation of strength. We measured RPR across 4,692 recombinant inbreds (RILs) comprising the maize nested association mapping (NAM) panel derived from crosses of diverse inbreds to the inbred, B73. An intermated B73×Mo17 family (IBM) of 196 RILs and a panel of 2,453 diverse inbreds from the North Central Regional Plant Introduction Station (NCRPIS) were also evaluated. We measured RPR in three environments. Family-nested QTL were identified by joint-linkage mapping in the NAM panel. We also performed a genome-wide association study (GWAS) and genomic best linear unbiased prediction (GBLUP) in each panel. Broad sense heritability computed on a line means basis was low for RPR. Only 8 of 26 families had a heritability above 0.20. The NCRPIS diversity panel had a heritability of 0.54. Across NAM and IBM families, 18 family-nested QTL and 141 significant GWAS associations were identified for RPR. Numerous weak associations were also found in the NCRPIS diversity panel. However, few were linked to loci involved in phenylpropanoid and cellulose synthesis or vegetative phase transition. Using an identity-by-state (IBS) relationship matrix estimated from 1.6 million single nucleotide polymorphisms (SNPs) and RPR measures from 20% of the NAM panel, genomic prediction by GBLUP explained 64±2% of variation in the remaining RILs. In the NCRPIS diversity panel, an IBS matrix estimated from 681,257 SNPs and RPR measures from 20% of the panel explained 33±3% of variation in the remaining inbreds. These results indicate the high genetic complexity of stalk strength and the potential for genomic prediction to hasten its improvement.

## Introduction

Maize stalk strength impacts grain yield and silage quality due to its relationship with stalk lodging and stover quality. High stalk strength is important in fields plagued by European corn borer, *Ostrinia nubilalis* H. [1], and Southwestern corn borer, *Diatraea grandiosella* D. [2]. Stalk strength also affects colonization of fungal pathogens such as *Gibberella zeae* [3] and *Diplodia zeae* [4]. High winds and soils with poor nitrogen to phosphorous ratios [5] increase stalk lodging in weak genotypes as well.

Dissection of stalk strength into its constituent traits suggests the structural composition of the rind, and not the pith or total girth, is the chief determinant of strength [6]–[8]. Previous study of maize rinds, from populations divergently selected for stalk strength, revealed several means for enhancement [6]. From anatomical analyses, increases in vascular bundles, rind-parenchyma inter-lumen thickness, and percent hypodermal cell wall area correlated with superior strength [6]. Vegetative phase change also occurred earlier in varieties with strong stalks [9]. In addition, compositional analyses have revealed the influence of cellulose and lignin on maize stalk strength [10].

Given the numerous mechanisms mediating stalk strength and the continuous variation observed for the trait, several studies were performed to quantitatively dissect its genetic architecture [11]–[14]. In the most extensive previous quantitative study of stalk strength, composite interval mapping of quantitative trait loci (QTL) controlling stalk strength was performed in four bi-parental maize families [14]. Construction of three of the families sought to maximize genetic variation for stalk strength by using parents divergently selected for stalk strength [14]. Stand counts and other metrics for stalk strength are environmentally dependent and not easily reproduced. Therefore, strength in the divergently selected parents was evaluated by stalk crushing strength and rind penetrometer resistance (RPR) [8], [14], [15]. RPR refers to the force required to pierce a stalk rind with a spike fixed to a digital force gauge [15], [16]. After parental selection, RPR was used to pierce the mid-internode below the primary ear. QTL controlling stalk strength were found in all four families [14]. Ear height was genetically correlated with RPR. Yet, most QTL remained significant after accounting for ear height variation [14].

Previous RPR studies laid a foundation for evaluating stalk strength. But, whole genome sequencing of B73 [17] and the construction of a maize HapMap detailing the segregation of millions of single nucleotide polymorphisms (SNPs) [18] now afford higher mapping resolution. For several traits, putatively causal alleles have been identified at the gene level in joint-linkage-assisted genome wide association studies (GWAS) [19]–[23]. Also, genomic best linear unbiased prediction (GBLUP) promises to increase breeding efficiency in complex traits. GBLUP employs all genotyped SNPs in estimation of an identity-by-state (IBS) genomic relationship matrix [24] and builds a linear mixed model from genotypic and phenotypic data. The model allows prediction of breeding values of genotyped seed before expending testing resources. This facilitates the selection of individuals with superior potential performance from a larger germplasm pool than what may be immediately field tested [25], [26].

Stalk strength remains a determinant of harvestable yield and forage quality in the use of maize for ruminant animals. Furthermore, new applications in cellulosic ethanol and biopolymer synthesis have caused a surge of interest in stalk strength related traits such as cell wall composition and biosynthesis [10]. While discoveries were made by molecular methods [10], there exists interest in quantitatively resolving the genetic architecture of natural variation in stalk strength. Leveraging our advanced knowledge of the maize genome as well as new mapping and prediction methods will enable us to understand the functional allelic diversity of stalk strength and to breed varieties that suit both enduring traditional needs and contemporary applications.

In this study, we measured the stalk strength (RPR), days to anthesis (DTA), and primary ear height (EHT) of approximately 200 recombinant inbreds (RILs) from each of 25 families composing the maize nested association mapping panel (NAM) [27]. An intermated B73×Mo17 family (IBM) of 196 RILs [28] and a diversity panel of 2,453 inbreds [29] collected from the North Central Regional Plant Introduction Station (NCRPIS) were also evaluated. We measured RPR across the NAM families in three environments. The IBM family and NCRPIS diversity panel were both measured in two environments. Bootstrapped joint-linkage QTL mapping [30] and a GWAS [19] were performed to resolve the genetic architecture of RPR, DTA, and EHT. Cross-validated family-nested QTL and GBLUP models were also constructed to assess the accuracy of predicting each line’s mean trait value across the surveyed environments [31].

## Materials and Methods

### Plant Materials and Environments

The NAM panel, developed by the Maize Diversity Project, was created as previously described [27]. In addition, 196 RILs of the IBM family [28] and 2,453 inbreds of the NCRPIS diversity panel [29] were surveyed. A total of 4,536 NAM and 174 IBM RILs were grown and measured at Muskgrave Research Station in Aurora, NY (silt-loam soil) in the summer of 2008. Plots of 4,471 NAM and 189 IBM RILs were measured in Rollins Bottoms Research Station in Columbia, MO (silt-loam soil) during the summer of 2009. In NY and MO environments, a single row plot was grown for each line. RILs were assigned to plots in a randomized fashion but stratified by family in both environments. B73 and the alternate parent of the NAM and IBM families were included in each block of 22 plots [32]. Plots were composed of 12 plants in NY and 20 in MO.

NAM families were also grown in Madison, WI at the Arlington Agricultural Research Station (silt-loam soil) in the summer of 2009. In WI fields, 3,453 RILs of the NAM panel were randomized and blocked into 10 maturity groups based on previous flowering data [30]. Each RIL was planted in a two-row plot containing 40 seeds per row. B73 and four replications of ten inbreds were randomly included as checks in each maturity group. All three environments were cultivated in a conventional manner with respect to fertilization, weed, and pest control. A total of 3,447 of the RILs were measured in all three environments and used for joint-linkage QTL mapping, GWAS, and genomic prediction (Table S1).

At Muskgrave Research Station in Aurora, NY (silt-loam soil) in the summer of 2010, a set of 2,293 NCRPIS inbreds were measured for RPR. The inbreds were planted in single row plots of 12 plants. The same year, 2,453 inbreds from the NCRPIS diversity panel were measured at South Farm Research Station in Columbia, MO (silt-loam soil). Single row plots of 15 plants were grown. B73, and replicates of the 25 alternate NAM parents, were randomly included as checks in the NY and MO field designs. The 2,293 inbreds measured in both environments were used in GWAS and genomic prediction of the NCRPIS diversity panel (Table S2).

### Phenotyping RPR and Related Traits

**Figure 1:**
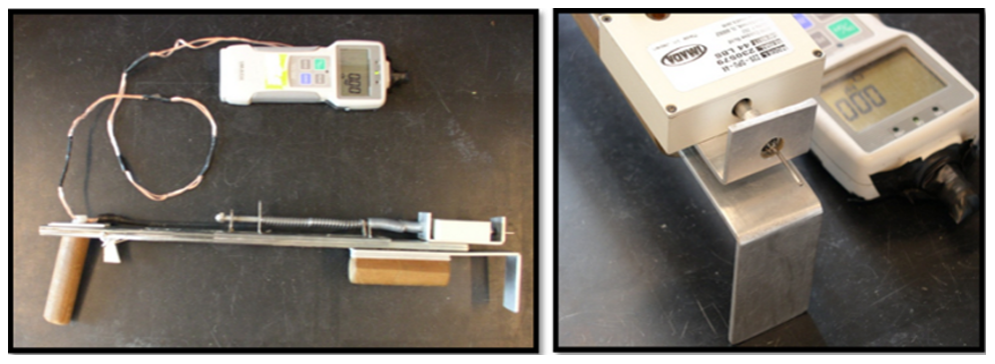
Spring-driven force gauge built to measure maize rind penetrometer resistance (RPR). A digital force gauge (model Z2S-DPU) measuring the kilograms of force imposed on a probe was adapted to accommodate a steel spike and fit to a triggered spring-driven track gliding on ball bearings. This apparatus was used to assess the force required to puncture maize stalk rinds with the spike mid-internode below the primary ear as a proxy for stalk strength. To ease data acquisition, custom Java code was developed to maintain measurements and provide users with audible commands denoting current inbred score and field plots remaining to be measured.

To measure stalk strength, a modified Accuforce Cadet digital force gauge (Ametek, Largo, FL) was assembled with a spike and used to manually pierce stalks as previously described [14] for RPR measures collected in Aurora, NY 2008. Subsequent measures were collected with a spring-driven Z2S-DPU digital force gauge reader (Imada, Northbrook, IL) to ease data collection (Figure 1). In this apparatus, a spike was fixed to the digital force gauge. The gauge was then fastened to a track and driven by release of a compressed spring. A trigger, cocking mechanism, and handle were fabricated to increase ease of measuring RPR by ensuring uniform acceleration of the spike, when driven into the rind. Custom Java code was developed to store plot mean RPR measures from the gauge for later analysis. Audible commands were encoded in the program to aid identification of plots measured and those remaining to be collected.

RPR measures were collected near the middle of the stalk internode immediately below the primary ear. Measures were taken from three randomly selected plants per plot. This resulted in the collection of 37,548 RPR measures across NAM families in three environments. Measures taken in the IBM family and the NCRPIS inbred diversity panel amounted to 1,735 and 15,399 observations, respectively. These were taken in two environments. To avoid differential nutrient availability and light capture, the end plants of each plot were not measured. Phenotypic data for DTA and EHT were acquired from the same plots as previously described [27], [30].

### Genotyping RIL Families and the NCRPIS Diversity Panel

Three molecular marker sets were used for joint-linkage QTL mapping, GWAS, and GBLUP across the NAM and IBM families, as well as linear mixed model GWAS and GBLUP across the NCRPIS inbred diversity panel. In the first marker set, 1,106 markers were genotyped on an Illumina Golden Gate assay across the RIL families to facilitate joint-linkage QTL mapping [27]. In this set, missing genotype calls were imputed as the weighted average of flanking markers. Weights were derived from the missing marker’s genetic distance to each adjacent marker as previously described [19].

A second marker set of 1.6 million SNPs reported in the maize HapMapV1 [18] were imputed across NAM family parents using fastPHASE v1.3 [33]. These SNPs were projected on the RIL families based on their parental lineage and the B73 genome as previously reported [19]. SNPs projected on the RILs were assigned a value equal to the weighted average of flanking markers from the first marker set of 1,106 markers genotyped across all RILs. Given a lack of recombination information, weights were estimated by a SNPs’ physical distance from flanking genotyped markers [19]. These SNPs were then used in joint-linkage-assisted GWAS and GBLUP of the RIL families.

A third marker set of 681,257 SNPs from the NCRPIS inbred diversity panel were genotyped using Genotyping-By-Sequencing (GBS) [34]. Missing SNPs were imputed by a nearest neighbors algorithm in TASSEL v.3.0 [35] and calculated from haplotypes constructed from SNPs in a surrounding window of 1,024 bp. SNPs were then used in linear mixed model GWAS of the NCRPIS inbred diversity panel and genomic prediction by GBLUP [31]. All three marker sets genotyped across the RIL families or the NCRPIS inbred diversity panel, and used in this study, are publicly available at www.panzea.org.

### Partitioning Phenotypic Variance and Estimating Line Means

To partition phenotypic variance into genetic and environmental components for RPR, DTA, and EHT, linear mixed modeling was performed using ASReml v3.0 [36]. We winsorized those plot observations greater than three standard deviations from each environmental mean, where environment denotes a unique field and year of measure. For each trait, linear mixed modeling was performed in coordination with custom Java code for bottom-up (variation in each environment was first fit and used to discern the terms included in the multi-environment likelihood function) backward selection of significant model terms based on likelihood ratio testing (p<0.05). Plot measures for the NAM and IBM families were fit in the same linear mixed model for each trait. However, measures for the NCRPIS inbred diversity panel were fit in a separate model given a lack of shared environments and few common lines.

For the RIL families, linear mixed models were first separately fit for each environment including a single fixed effect for the grand mean and multiple random effects. Random effects entering the full model included family and RIL nested within family genotypic effects, as well as the environmental effects of the blocks, rows, and columns of each field design. A separate variance component was fit for RIL nested within each of the NAM and IBM families. An additional family term was constructed to accommodate the replicated parental checks. All random effect terms in the model, including the genotypic effects of family and RIL within family, were modeled with independent G structures. However, a correlated R structure was fit among residuals based on a two-dimensional separable first-order autoregressive spatial structure for rows and columns in each environment (AR1_row_ ∶ AR1_col_). All environmental effects and residual correlation structures in each environment’s model were tested by backward selection and retained if they met a likelihood ratio significance of p<0.05. BLUPs for each RIL in the NAM and IBM families were then predicted in each environment.

Next, for each trait a single linear mixed model across environments was fit for the RIL families, including and nesting the significant components of each individual environment model by environment. Across environment terms were also added. These included environment, family-by-environment, and RIL nested within family-by-environment interaction terms, as well as a heterogeneous spatially correlated R structure across environments, maintaining significant autoregressive spatial structures for rows and columns in each environment. BLUP line means for each RIL were predicted from the multi-environment model for use in mapping and prediction (Table S1). After fitting the multi-environment model for RPR, a random effect for DTA variation was included in an additional model to evaluate the variance captured by genotypic and environmental effects after accounting for DTA. Analogously, EHT was included in another model to assess its effect on RPR variance explained by genotypic and environmental effects.

In the NCRPIS inbred diversity panel, a single linear mixed model was fit across both environments including a single fixed effect for the grand mean and random effects for genotypic and environmental factors using ASReml v3.0 [36]. Random effects entering the full model included an inbred genotypic effect and the environmental effects of field, row, column, and blocks within each environment. As in the case of NAM and IBM families, all random effect terms including genetic effects were modeled with independent G structures. A two-dimensional separable first-order autoregressive spatial structure for rows and columns in each environment and a heterogeneous correlated covariance structure across environments were modeled in the R structure of the residuals. From the multi-environment model constructed for each trait, BLUP inbred line means in the NCRPIS diversity panel were estimated for use in mapping and prediction (Table S2). After fitting this full model, random effects for DTA and EHT were also sequentially included in additional RPR models to assess their influence on the variance in RPR captured by genotypic and environmental effects.

As a result of fitting a separate variance component for RIL nested within each NAM and IBM family as well as among the replicated parental checks, differing levels of shrinkage among BLUP line means for the three traits existed in each of the RIL families. Therefore, models constructed across and within environments were refit including the family term and all RIL nested within family terms as fixed, rather than random effects. This enabled comparison of best linear unbiased estimated (BLUE) line means for these traits (Table S1). The unbiased distribution of line means in each family enabled comparisons to replicated parental values and improved inference of their relative rank across families. The mixed model constructed for the NCRPIS diversity panel was refit with fixed genetic effects in an analogous fashion (Table S2).

### Calculating Broad Sense Heritability on a Line Means Basis

For each trait, estimates of broad sense heritability on a line means basis across and within RIL families were calculated from the multi-environment models fitting genotypic effects as a random effect, as previously described [32]. The variance component explaining variation between NAM families and the arithmetic mean of the 25 variance components explaining variation between RILs within each of the families were summed to infer the genetic variance between RILs across families for each trait. The genetic variances in each NAM family and the IBM family were estimated as the variance component explaining variation between RILs within that family. These estimates of between RIL variance composed the numerator of the broad sense heritability estimator, when computed on a line means basis, across and within NAM and IBM families, respectively.

The denominator of the broad sense heritability estimator, when computed on a line means basis [32], reflects the total variation between and within RILs, less any environmental or covarying trait (RPR models possessing DTA or EHT covariates) variance ascribed to alternate sources of variation in the model. It is the sum of the numerator and that of the estimated contributions of family-by-environment, RIL nested within family-by-environment, and residual error to the expected variation in a single RIL. To estimate these contributions to a single RIL in an unbalanced design, family-by-environment and RIL nested in family-by-environment were divided by the harmonic mean of the number of environments in which each family or RIL was measured. The contributions of residual error to variation in a RIL was estimated by dividing the arithmetic mean for error across the heterogeneously modeled residuals for each environment by the harmonic mean of the number of plots a RIL was estimated. Broad sense heritability on a line means basis in the NCRPIS diversity panel was calculated in a similar manner [32]. However, no family or family-by-environment terms were included during estimation of the genetic variation existing between or within the inbred lines.

### Bootstrapped joint-linkage QTL Mapping across RIL Families

To map genetic associations underlying the estimated heritable variation, the SAS v9.2 statistics package [37] was implemented. SAS PROC GLMSelect was applied to regress BLUP line means for RPR, DTA, and EHT against a family term as well as a subset of 1,106 markers nested within each of the NAM and IBM families in a family-nested QTL model. First, a model term was fit for each of the 26 RIL families. Subsequently family-nested marker selection was performed by stepwise regression [30]. For all traits, the significance of model entry and exit, p<5e-4, was obtained for the marginal F-test of a family-nested marker, based on the results of permutation testing.

Next, the previously described stepwise joint-linkage mapping procedure [30] was bootstrapped. For 100 samples, a random 75% of the RILs stratified within each of the families were selected without replacement and a random sampling of 25% of the RILs within each family were selected with replacement followed by family-nested QTL selection. This led to the construction of 100 family-nested QTL models. To rank their robustness, a resample model inclusion probability (RMIP) [38] was also calculated for family-nested QTL (Table S3). A RMIP details the probability a family-nested QTL was included in a model of genetic architecture upon family-stratified bootstrapping of the full data set. Upon permutation testing of RPR, DTA, and EHT, a type 1 error rate <0.05 was identified for family-nested QTL with an RMIP of over 10 out of 100 models at the given model entry and exit criteria, p<5e-4. SAS code detailing the RIL family-stratified bootstrapping of family-nested QTL mapping is available upon request.

After constructing a family-nested QTL model for RPR, DTA, and EHT from the full dataset, each trait’s model was fit to every other trait for estimation of pleiotropic family-nested QTL. Significance of pleiotropy was determined as previously described [30]. It was based upon the significance of correlation between the estimated allele effects for each trait at a given family-nested QTL across all 26 RIL families.

### GWAS across RIL Families and the NCRPIS Diversity Panel

By modifying family-nested QTL models for RPR, DTA, and EHT constructed from the full dataset, ten additional models, one for each maize chromosome, were built to account for background genetic variation during joint-linkage-assisted GWAS. In the family-nested QTL models, all terms explaining significant trait variation were fit, with the exception of the family term denoting variation between the NAM and IBM families. Family-nested QTL residing on a model’s designated chromosome were also dropped. Residual trait variance after fitting this model was inferred to represent the joint distribution of error and the missing family-nested QTL of the dropped chromosome.

For each trait, residuals from each of the ten chromosome’s models were used in joint-linkage-assisted GWAS of the HapMapV1 SNPs [19]. To perform joint-linkage-assisted GWAS, model residuals were regressed against SNPs of their respective chromosome in a bootstrapped forward regression procedure as previously described [19]–[21]. A family term for variation between RIL families was fit in the model prior to marker selection. The threshold for model entry was set to p<5e-8 by permutation testing. A total of 100 sampling iterations of a random 75% of the inbreds without replacement were followed by SNP selection to attain an estimate of a SNP’s RMIP [38] and assess the robustness of observed associations (Table S4). Unlike joint-linkage QTL mapping, this approach did not nest SNPs in each RIL family. Upon permutation testing of RPR, DTA, and EHT, SNPs with a RMIP greater than 5 out of 100 models possessed a type I error rate less than 0.05 at the model entry/exit criteria of p<5e-8.

Using the Genome Association and Prediction Integrated Tool (GAPIT) [39] in R v2.12.0 [34], sequential single SNP linear mixed model GWAS was performed for RPR BLUP line means in the NCRPIS diversity panel (Table S5). This approach accounted for population structure by inclusion of an identity-by-state (IBS) genomic relationship matrix and allowed the identification of associations with RPR [40]. The IBS relationship matrix was constructed by applying the Van Raden method [24] to 681,257 standardized SNPs genotyped in the NCRPIS diversity panel. Using GAPIT, association significance was determined by estimates of false discovery rate, estimated using the Benjamini-Hochberg method, across all GWAS tests performed for RPR in the NCRPIS diversity panel [41]. The proximity of established candidate genes to identified associations was inferred based on functional annotations of the maize genome sequence release 4a.53.

### Cross-validated Genomic Prediction across RIL Families and the NCRPIS Diversity Panel

We conducted genomic prediction by GBLUP using the package rrBLUP [31] in R v2.12.0 [34]. IBS genomic relationship matrices [24] were constructed using the Van Raden method [24] for the NAM and IBM families from 1.6 million standardized SNPs of the maize HapMapV1 [18]. Estimates of genomic relationship among the NCRPIS diversity panel were calculated using the 681,257 standardized SNPs [35]. BLUP and BLUE line means for RPR, DTA, and EHT estimated across environments in a first stage model were regressed against the IBS genomic relationship matrices of the RIL families and NCRPIS diversity panel to obtain genomic BLUPs for each inbred and trait.

To perform cross-validation, all NAM RILs (not stratified by family), each NAM and IBM family, and the NCRPIS diversity panel were randomly partitioned into five disjoint subsets. Line means from combinations of one (5 subsets choose 1 calibration set  = 5 folds), two (10 folds), three (10 folds), and four (5 folds) subsets were used to calibrate models and predict the remaining line mean values. All prediction accuracies were then averaged across folds. This process was repeated 20 times randomly selecting five disjoint subsets each time to estimate prediction accuracy with respect to the number of lines in the model calibration set.

For comparison to GBLUP, prediction of RPR BLUP line means by joint-linkage mapping of family-nested QTL was also performed using PROC GLMSelect in the SAS v9.2 statistics package [37]. However, unlike the bootstrapping approach taken to estimate robust family-nested QTL, cross-validation was performed using family-nested QTL models constructed from disjoint subsets of the RILs to predict the remaining RILs. This was performed in a sampling scheme identical to family-stratified sampling of the NAM panel during GBLUP. A family term was included in every model and the thresholds for model entry/exit were relaxed to p<0.05. The significance of marker inclusion was not tested by permutation as in the bootstrapping approach; however, a maximum of 40 family-nested QTL were permitted inclusion in the model.

Prediction accuracy was measured as the coefficient of determination obtained by regressing BLUP or BLUE line means from first stage models against predicted line means obtained by GBLUP or the family-nested QTL models of second stage models for each trait upon cross-validation. This differs from traditional estimates of genomic prediction accuracy as the coefficient of determination between genomic estimated and true breeding values. This metric was not calculable given a lack of true breeding values for RPR in RIL families or the NCRPIS inbred diversity panel.

## Results

### Variation in RPR and Related Traits

**Figure 2:**
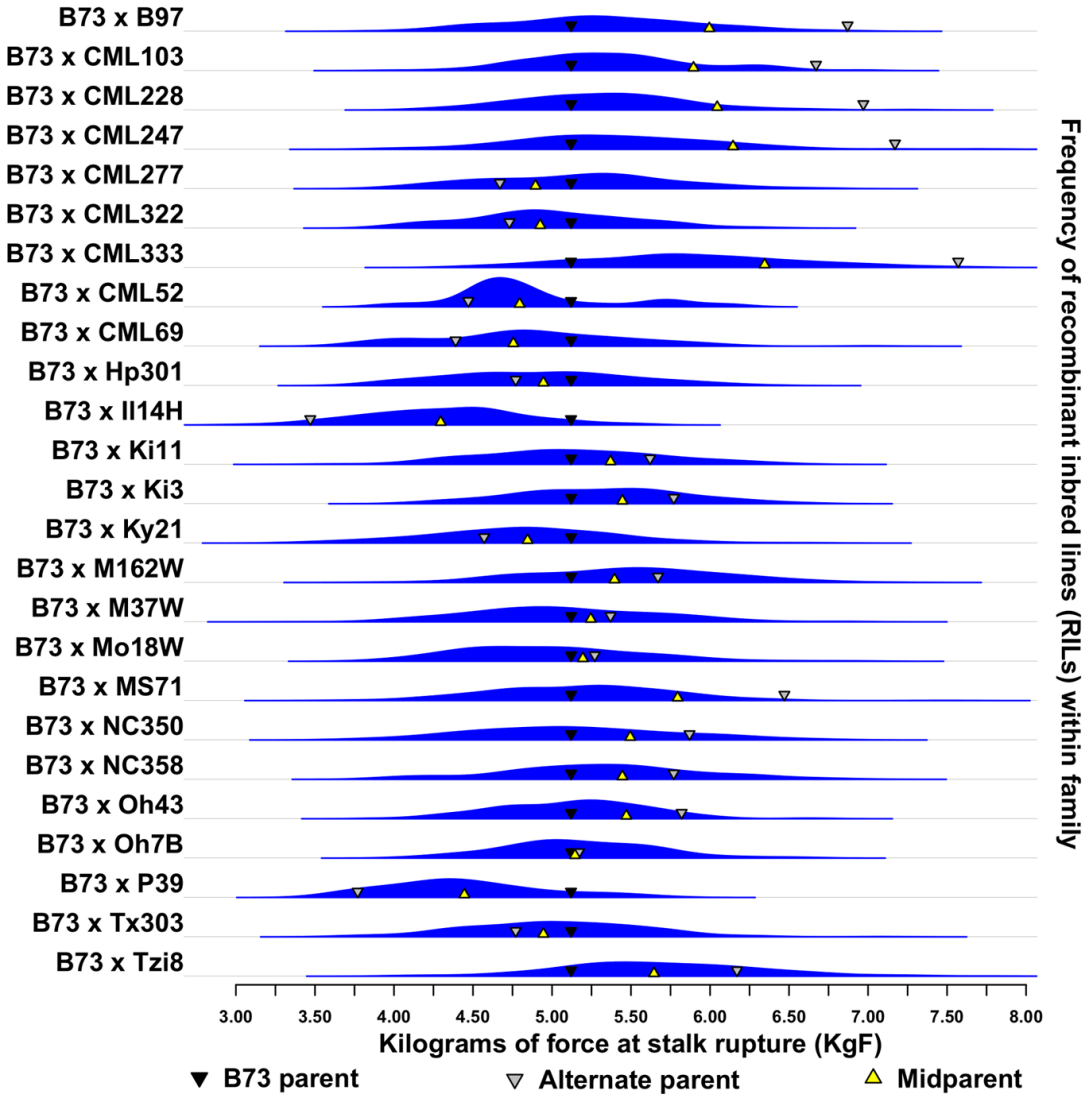
Asymmetric transgressive segregation observed for RPR. Comparisons of parent, mid-parent, and progeny BLUE line means for RPR revealed transgressive segregation. Parents of the NAM and IBM families were not chosen from populations divergently selected for RPR. Recombination of additive effects and novel mutations likely play a role in the transgressive variation among their inbred progeny. Furthermore, the asymmetry present in these distributions suggests a role for epistasis.

Heritable variation between maize lines was identified for RPR, EHT, and DTA within the NAM, and IBM families, as well as the NCRPIS inbred diversity panel (Table 1). Transgressive segregation of the traits was also identified in most RIL families, based on comparison of parental BLUE line means and their RIL progeny (Figure 2). However, in the case of RPR, few contrasts were statistically significant. BLUE values for inbreds revealed 95% of the RILs fell between 4.03 and 6.84 kilograms of force (KgF). In the NCRPIS diversity panel, 95% of RPR measures ranged from 2.66 KgF to 10.02 KgF. Substantial variation for DTA and EHT also existed within this panel (Figure S1). Based on review of line means for RPR across replicated NAM progenitors, the inbreds with the weakest RPR among the NAM and IBM family parents were the sweet corn inbreds, IL14H and P39. The common parent, B73 had a mean RPR of 5.18 KgF and was stronger than one third of the NCRPIS diversity panel. It also had a higher RPR than nine of the 25 alternate NAM parents (p<0.1). The BLUE line mean of B73 for RPR was weaker than Mo17, the alternate parent of the IBM population. However, this contrast was not statistically significant.

**Table 1:** Broad-sense heritability of RIL families and the NCRPIS diversity panel.

Family/Panel	Plants scored	RPR	RPR (DTA cov)	RPR (EHT cov)	DTA	EHT
**NAM panel**	37,548	0.21	0.20	0.21	0.94	0.93
**B73×B97**	1,763	0.20	0.20	0.20	0.85	0.94
**B73×CML103**	1,804	0.07	0.06	0.06	0.85	0.95
**B73×CML228**	1,211	0.07	0.08	0.07	0.94	0.93
**B73×CML247**	1,313	0.28	0.29	0.26	0.93	0.93
**B73×CML277**	1,311	0.16	0.16	0.15	0.94	0.93
**B73×CML322**	1,530	0.03	0.04	0.02	0.92	0.92
**B73×CML333**	1,554	0.33	0.31	0.30	0.94	0.93
**B73×CML52**	1,105	0.03	0.04	0.03	0.95	0.92
**B73×CML69**	1,378	0.19	0.20	0.18	0.89	0.93
**B73×Hp301**	1,815	0.11	0.12	0.10	0.90	0.95
**B73×Il14H**	1,587	0.05	0.05	0.04	0.91	0.93
**B73×Ki11**	1,352	0.12	0.11	0.12	0.94	0.94
**B73×Ki3**	997	0.04	0.03	0.03	0.93	0.92
**B73×Ky21**	1,611	0.17	0.18	0.17	0.84	0.93
**B73×M162W**	1,556	0.15	0.14	0.14	0.91	0.92
**B73×M37W**	1,641	0.15	0.14	0.13	0.90	0.91
**B73×Mo18W**	1,365	0.08	0.08	0.07	0.93	0.93
**B73×MS71**	1,684	0.08	0.09	0.08	0.78	0.90
**B73×NC350**	1,517	0.28	0.24	0.26	0.92	0.93
**B73×NC358**	1,685	0.24	0.25	0.23	0.84	0.92
**B73×Oh43**	1,715	0.24	0.21	0.21	0.81	0.93
**B73×Oh7B**	1,548	0.03	0.03	0.03	0.90	0.95
**B73×P39**	1,473	0.02	0.04	0.01	0.95	0.93
**B73×Tx303**	1,505	0.03	0.02	0.02	0.92	0.95
**B73×Tzi8**	1,526	0.28	0.24	0.25	0.93	0.95
**B73×Mo17(IBM)**	1,735	0.34	0.30	0.31	0.92	0.96
**NCRPIS panel**	15,399	0.54	0.63	0.65	0.92	0.86

All reported broad-sense heritability estimates are calculated on a line means basis. RPR, DTA, and EHT detail the proportion of variance between and within lines explained by between line variance after accounting for known environmental variance in the trait. RPR (DTA cov) and RPR (EHT cov) detail the proportion of variance between and within lines explained by between line variance after accounting for known environmental variance and the covariance of RPR with DTA and EHT, respectively.

Broad sense line means heritability estimates for RPR measures in the surveyed germplasm were low (Table 1). This was true both across NAM families where the broad sense heritability estimated on a line mean basis was 0.21, and within NAM families where the average broad sense heritability was 0.17 when estimated on a line means basis. Of the 25 NAM families, 19 families possessed an estimated broad sense heritability for RPR above 0.05. At a broad heritability of 0.34, a higher proportion of RPR variation was heritable in the IBM family than among any of the NAM families studied. RPR in the NCRPIS diversity panel was more heritable with a broad sense line means heritability of 0.54. Accounting for covariation of DTA and EHT with RPR in the NCRPIS diversity panel increased broad sense heritability estimates to 0.63 and 0.65, respectively. In contrast, accounting for this covariation in NAM and IBM families did not greatly influence estimates of broad sense heritability in RPR across or within families.

**Figure 3:**
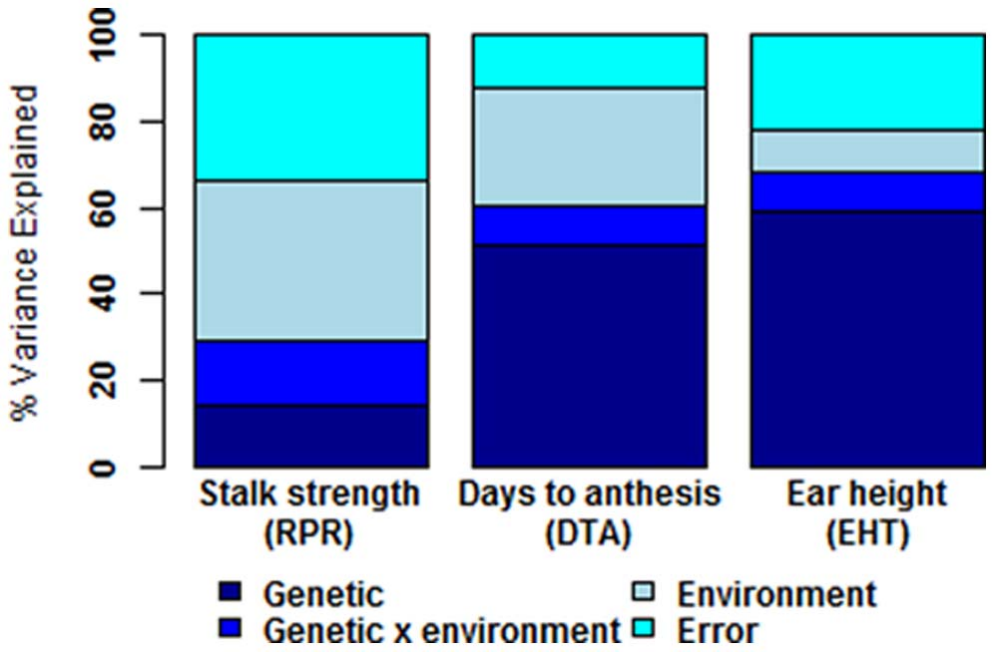
Variation in RPR, days to anthesis (DTA), and ear height (EHT). About 15% of the total variation in RPR across all NAM and IBM families was attributable to genetic factors. This proportion of genetic variation was smaller than that observed for DTA or EHT. Despite this reduction in genetic variation of RPR, the proportion of genetic-by-environment variation were slightly larger for RPR than the other surveyed traits. Differences in the remaining RPR, DTA, and EHT variation were due to environmental factors or could not be attributed to known sources of variation.

About 37% of stalk strength variation in the NAM panel was attributed to environmental variation (Figure 3). This was greater than that observed for DTA or EHT. Nonetheless, measures of environmental variation were confounded with manual and spring-driven RPR phenotyping methods. Estimates of the proportion of genotype-by-environment variation in RPR were about 11%, but were also confounded by phenotyping method. Correlations between BLUE line means for RPR in the RIL families calculated using the manual RPR approach, taken in New York 2008, and spring-driven RPR approach, taken in Missouri (r  = 0.37, p<5e-3) and Wisconsin (r = 0.20, p<5e-3), were weaker than correlation between the spring-driven RPR environments (r  = 0.51, p<5e-3).

**Figure 4:**
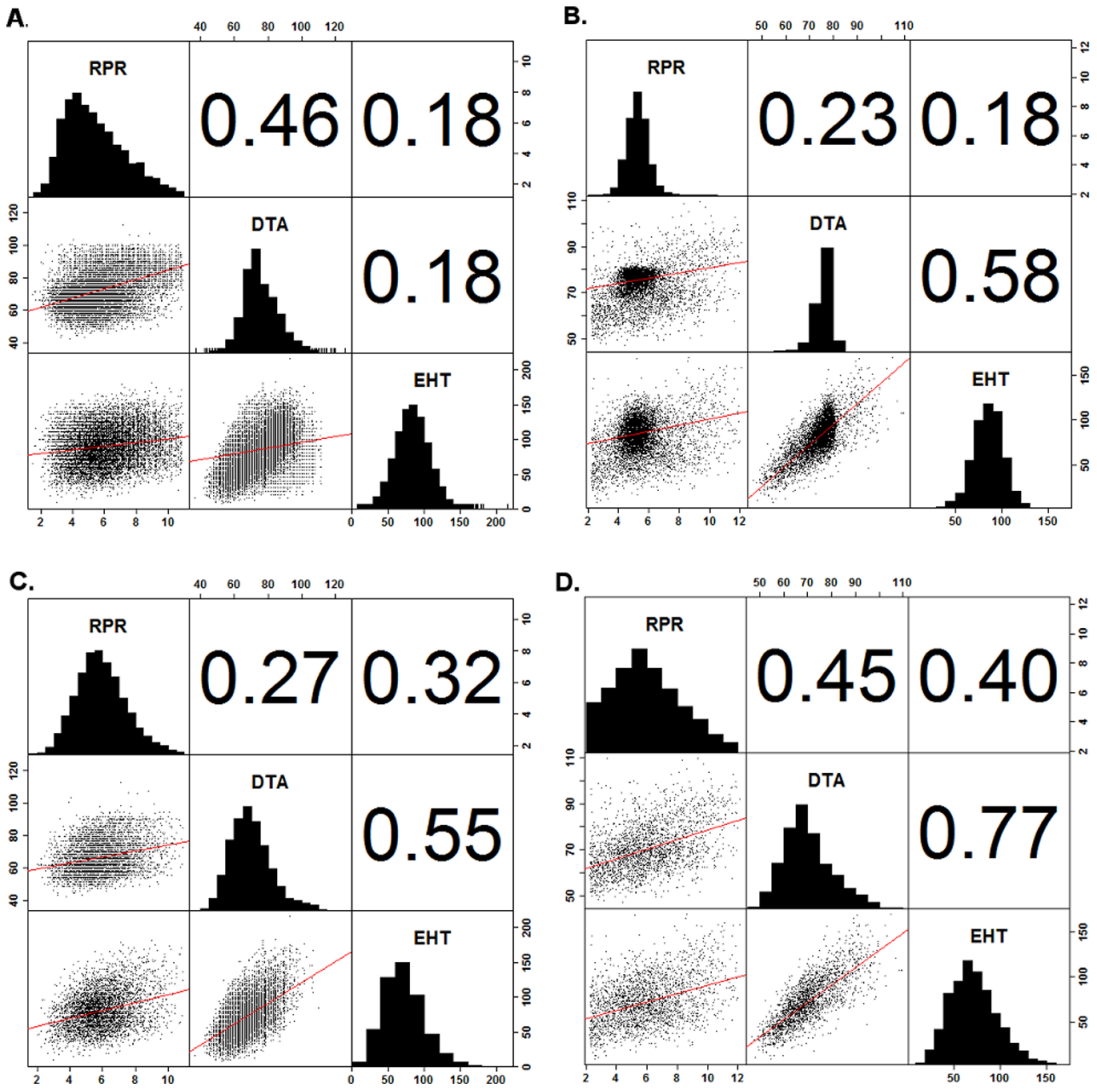
Trait correlations between RPR, DTA, and EHT variation. Positive correlations between RPR and DTA were greater among plot means (A) than line means (B) across all RILs of the NAM families. However, the opposite relationship was observed between these traits among plot means (C) and line means (D) in the inbreds of the NCRPIS diversity panel. The relationship between RPR and EHT was less varied between the NAM and NCRPIS diversity panels.

Correlations between RPR and DTA, were positive among plots across NAM and IBM families (r  = 0.46, p<5e-3, Figure 4A) and within many of the families (Figure S2). However, correlations among BLUE line means across RIL families were reduced (r  = 0.23, p<5e-3, Figure 4B). In the NCRPIS diversity panel, the opposite trend was found between RPR and DTA among plots (r  = 0.27, p<5e-4, Figure 4C), and the BLUE line means (r  = 0.45, p<5e-3, Figure 4D). RPR and EHT correlations were weakly positive among plots across RIL families (r  = 0.18, p<5e-3, Figure 4A); but, varied widely within families (Figure S2). Correlations among the BLUE line means were similar (r  = 0.18, p<5e-4, Figure 4B). In the NCRPIS, these correlation were similar among plot measures (r  = 0.32, p<5e-3, Figure 4C, Figure S2), and BLUE line means (r  = 0.40, p<5e-3, Figure 4D, Figure S2).

### Joint-linkage QTL Mapping of RPR and Related Traits in RIL Families

Using bootstrapped joint-linkage mapping, family-nested QTL explaining variation in BLUP inbred line means for RPR were detected across NAM and IBM families on all chromosomes (Table 2, Figure S3). Differences between families explained 60±4% of the variation in BLUP line means for RPR in the NAM and IBM families. But, inclusion of family-nested QTL brought the fraction of RPR variation explained up to 81±3%. A total of 78 of the 1,106 family-nested markers possessed a RMIP greater than 10 (Table S3, Figure S3). However, only 18±2 family-nested QTL were included within any given model build constructed from a single bootstrap sample before reaching the model entry threshold set by null permutation.

**Table 2:** Family-nested QTL for RPR in RIL families.

Chr	Mb	cM	RPR (RMIP)	DTA (RMIP)	EHT (RMIP)	Local annotations of interest
1	15.7	31.7	31	59	10	
1	22.3	39.2	34	22	26	
1	34.4	54	22	5	0	
1	251.1	146.6	28	9	66	
1	260.3	156.5	27	15	4	
1	281.8	176.9	26	15	15	
1	285.9	180.9	20	61	13	
2	27.8	58.8	29	3	47	
2	31.4	62.2	35	13	6	
2	161.8	82.5	24	25	14	*Cellulose Synthase-9* (GRMZM2G018241)
2	192.6	103.7	31	21	14	
2	233.1	155.7	36	15	13	
3	5.2	20.1	37	11	15	
3	28.0	53.4	20	65	37	
3	178.2	90.0	35	6	62	
4	241.7	126.9	28	5	13	
5	5.9	22.1	18	0	0	
5	75.2	66.8	25	54	57	
5	175.5	87.2	23	3	1	
5	211.9	138.0	18	8	8	
7	6.0	26.3	18	18	0	
7	159.4	105.2	61	2	3	
7	170.0	135.0	44	78	7	
8	163.1	94.1	53	45	11	
8	164.8	100.3	40	15	4	
9	4.0	0.0	23	2	2	
9	16.2	28.5	38	3	6	
9	25.7	42.8	20	31	32	
9	140.9	85.6	17	24	7	
9	148.7	107.6	19	0	1	
9	150.1	114.8	19	3	1	
10	77.5	38.6	31	34	64	Predicted cellulose synthase activity by homology (GRMZM2G157729, GRMZM2G110145)
10	139.6	69.2	19	6	5	Putative 4-coumarate-CoA ligase-like gene (AF466202.2_FG012)

The reported resample model inclusion probabilities (RMIP) of this table detail the number of models one or more markers located within three cM of the noted association was selected out of 100 models. Each of these models was constructed from family-stratified sampling of the RILs with replacement. Physical positions are stated with respect to reference genome AGPv1.

At a RMIP of 61, the most robust family-nested marker association was located on chromosome seven at about 105.2 cM on the composite NAM map [27]. Separated by about 1.7 cM, two neighboring markers on chromosome eight located at about 97.4 cM possessed RMIPs of 35 and 28. A strong positive correlation (r  = 0.78, p<5e-3) of median allele effects across NAM and IBM families for these markers suggested the segregation of a common factor.

Partitioning variation in BLUP line means for RPR revealed no single family-nested marker association explained over 2.7% of the within family variation. Allele effects of the joint-linkage marker associations were small across families (Table S3). While 95% of effect estimates in the NAM and IBM families spanned a range of 1.22 KgF, the median significant (T-test allele effect within family, p<5e-3) negative and positive effects across the 100 model builds were comparable and about 0.07±0.01 KgF from the family mean of the BLUP line means for RPR. The number of associations found in a family was correlated with that family’s heritability (r  = 0.89, p<5e-3). The median number of families in which a marker possessed a significant effect for RPR was 9±3 out of the 26 families and was correlated with its RMIP (r  = 0.81, p<5e-3).

B73 and all 26 alternate parental inbreds of the NAM and IBM families had positive and negative effects for RPR across the 78 joint-linkage mapped associations. Given the weak correlations among inbreds for RPR, DTA and EHT, no significant correlation of allele effect estimates across NAM and IBM families were observed, when DTA and EHT were regressed against the RPR constructed family-nested QTL model built from the full dataset. Similarly, no significant correlation of RPR allele effects estimated were identified when RPR was regressed against the DTA and EHT constructed family-nested QTL models built from the full dataset. Nonetheless, weak correlations between RPR and both DTA (r  = 0.31, p = .12), and EHT (r  = 0.30, p = .12), were observed across the NAM and IBM BLUP family means.

The resolution afforded by joint-linkage mapping did not provide gene level characterization of RPR associations. In most instances, robust family-nested marker association persisted an interval of one to three cM before dropping below a RMIP of 10. In these intervals, few genes with known involvement in phenylpropanoid or cellulose synthesis pathways were identified. While no robust associations were found near the *brown midrib* mutants involved in lignin biosynthesis of the phenylpropanoid pathway, a putative 4-coumarate-CoA ligase-like gene (AF466202.2_FG012) potentially involved in the same pathway was located near a linkage marker on chromosome ten at about 69.2 cM with a RMIP of 18. Also, a caffeoyl-CoA O-methyltransferase (GRMZM2G077486) of the phenylpropanoid pathway was flanked by linkage markers on chromosome ten at 38.6 and 40.1 cM possessing RMIPs for RPR of 20 and 11, respectively.

Of the 12 known cellulose synthases in the maize genome, the only synthase whose nearest linkage marker possessed a RMIP over 10 was cellulose synthase-9 (GRMZM2G018241). This gene and marker are located on chromosome two at 82.5 cM and possessed a RMIP of 24. Uncharacterized annotations (GRMZM2G157729, GRMZM2G110145) with predicted cellulose synthase activity by homology and known transcriptional evidence were also identified on chromosome nine near a linkage marker at about 42.8 cM and chromosome ten at about 38.6 cM on the NAM composite map. Both these linkage markers possessed a RMIP of 20, with the latter also flanked by a marker possessing a RMIP of 10. Cloned loci previously identified for vegetative phase transition and other stalk strength related traits, such as the mutants of *brittle stalk 2, glossy1–15,* and *teopod1, 2* were not identified near associations possessing RMIPs for RPR over 10. Similarly, co-localization of QTL mapped in the previous multi-family RPR study and this family-nested QTL study was not substantial [14].

### GWAS of RPR and Related Traits

To further resolve joint-linkage mapped RPR associations, 141 significant associations were identified by joint-linkage-assisted GWAS in the NAM and IBM families (Figure S3, Table S4). Family-nested QTL identified during joint-linkage mapping were used to account for background genetic variation during GWAS. The most robust GWAS associations co-localized with estimated joint-linkage mapped effects. However, many significant effects were found across the maize genome. No significant RPR associated SNPs were shared with joint-linkage assisted GWAS of DTA (277) or EHT (304) in the NAM and IBM families. About 5% (15) of DTA associations were located within 1 cM of a RPR association; whereas, about 10% (29) of the EHT associations were located within 1 cM of an RPR association. Nearly one third (43) of associated SNPs were identified in a known or hypothesized gene. However, no significant associations were located in genes with known involvement in the phenylpropanoid or cellulose synthesis pathways (Table S4). Also, no associations with a RMIP over three were identified in 100 kb of genes with established involvement in these pathways. The same was true of genes implicated in vegetative phase transition.

The effect sizes of GWAS RPR associations across the NAM and IBM families were uniformly small and similar in size and distribution to the significant alleles nested within each family during joint-linkage QTL mapping. No RPR effects with an absolute value greater than 0.05±0.02 KgF were observed for GWAS effect estimates. The most robust association identified across the NAM and IBM families possessed a RMIP of 100. This SNP is located on chromosome three at 176,660,475 bp and was flanked by linkage markers that possessed RMIPs of 10 and 13 during joint-linkage mapping. The nearest annotation is 5,139 bp downstream and encodes a transferase (GRMZM2G165192) responsible for transferring acyl groups other than amino-acyl. No annotations within a 1 cM interval surrounding the association were obvious candidates for stalk strength. The second and third most robust associations were both identified on chromosome eight at 163,943,201 bp and 8,415,595 bp. These possessed RMIP of 94 and 73, respectively. Both were also located near regions of the genome neighboring significant markers identified in joint-linkage analysis. The former is located in an interval wherein two linked markers spaced about 1.7 cM apart possessed a combined RMIP of 62. The latter neighbors a linked marker with a RMIP of 14 in joint-linkage mapping. In both instances, no obvious candidates for stalk strength related pathways or developmental processes were apparent. The nearest respective annotations were a glycosyl-transferase (GRMZM2G002023) 4,605 bp downstream and an O-glycosyl hydrolyzing enzyme (AC234160.1_FG003) 3,349 bp downstream.

In addition to joint-linkage-assisted GWAS across the NAM and IBM families, sequential single marker GWAS was also performed across the NCRPIS diversity panel regressing BLUP line means for RPR against SNPs. (Figure S3, Table S5). To account for the population structure of the panel that was not reduced by the recent recombination of genetic diversity a linear mixed model framework including a term for kinship was implemented [42]. Using this method to query 681,257 GBS SNPs, the most significant RPR associations still possessed a false discovery rate above 10%. Upon review of the top 500 associations possessing a false discovery rate less than 17%, the most significant explained less than 1% of the heritable variation of RPR in the NCRPIS diversity panel. The median minor allele frequency among these top 500 enriched associations was 2.1%. None of the enriched associations in the NAM and IBM families or the NCRPIS diversity panel were identified in the *brown midrib, brittle stalk,* or *teopod* loci or near enzymes with known involvement in phenylpropanoid and cellulose synthesis pathways or vegetative phase transition. Comparisons between significant RPR associations identified in the RIL families and those enriched in the NCRPIS inbred diversity panel revealed little discernible commonality.

### Genomic Prediction of RPR and Related Traits

**Figure 5:**
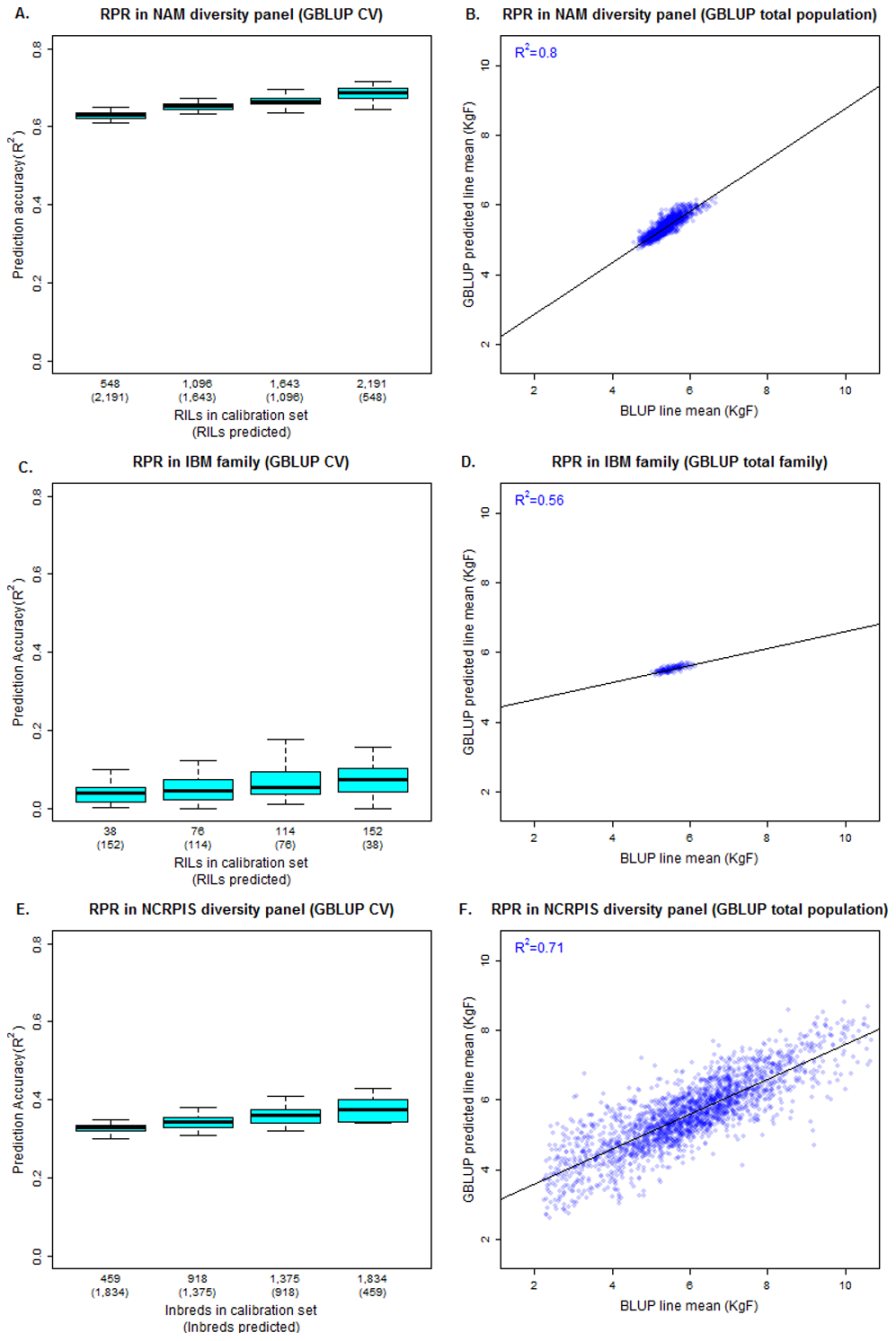
Prediction of RPR in RIL families and NCRPIS diversity panel by GBLUP. Cross-validation revealed steady gains in prediction accuracy with respect to the NAM family-stratified calibration set size (A). About 80% of the variation in BLUP line means for RPR was explained using an identity-by-state (IBS) genomic relationship matrix constructed from 1.6 million SNPs of the maize HapMapV1 (B). Using the same SNP set, prediction accuracy was not significant in the IBM family (C), and the variation explained by the entire family was only 56% (D). In the NCRPIS diversity panel, prediction accuracies were lower than in the NAM panel when using an IBS genomic relationship matrix of 681,257 SNPs constructed and fit in the same manner (E). In total, 71% of the variation in RPR was explained upon fitting the entire panel (F).

Given the highly polygenic architecture of RPR, genomic prediction was performed to determine the ability of all genotyped diversity to explain variation in BLUP line means for RPR, DTA, and EHT across all NAM families possessing a broad sense line means heritability over 0.05 (Figure 5, Figure S4). An IBS genomic relationship matrix was constructed from 1.6 million maize HapMapV1 SNPs and fit in a GBLUP framework [24], [31]. Cross-validation revealed strong prediction accuracy, 20% of the RILs (approximately 40 lines), randomly but equally chosen from each heritable NAM family, calibrated a model predicting 64±2% of BLUP line mean variation in RPR among the remaining RILs (Figure 5A). When 80% of the RILs (approximately 160 lines from each family) were used to calibrate the model, the variation in RPR explained increased to 68±4%. Prediction accuracies did not significantly differ when RILs chosen for the calibration set were not stratified by family. When all RILs from all heritable families were included in model calibration, 80% of the BLUP line mean variation in RPR was explained by the IBS genomic relationship matrix (Figure 5B).

Fitting a family-nested QTL model with an included family term, constructed by joint-linkage mapping of calibration sets subsampled from each of the heritable NAM families, wielded lower prediction accuracies than GBLUP (Figure S5A). Constructing a family-nested QTL model using 20% of the RILs in each NAM family predicted 28±7% of BLUP line mean variation in RPR among the remaining lines. Falling short of the prediction accuracy achieved by GBLUP, using 80% of the RILs in each family only increased the prediction accuracy to 40±14% of BLUP line mean variation in RPR among the remaining lines. At every calibration set size, variation in prediction accuracy was larger between calibration sets than that observed in GBLUP. However, when all RILs were included in joint-linkage mapping across the NAM panel, 81% of the BLUP line mean variation in RPR was explained, a value comparable to that attained by GBLUP (Figure S5B).

An IBS relationship matrix constructed for the RILs of the IBM family was less predictive of variation in BLUP line means for RPR, DTA, and EHT than that obtained across all NAM families when fit by GBLUP (Figure 5, Figure S4). Cross-validation revealed about 20% of the RILs (38 lines) could not predict BLUP line means of RPR in the remaining lines (Figure 5C). This did not substantially improve as 80% (152 lines) of the RILs in the IBM family were used to predict the remaining line means and explained 8±7% of their variation. In contrast, approximately 56% of the BLUP line mean variation in RPR was explained by a GBLUP model constructed from the entire IBM family (Figure 5D). Comparable levels of variation in BLUP line means for RPR, DTA, and EHT to that explained in the IBM family were explained in predicting and fitting most of the similarly sized heritable NAM families.

In addition to RIL families, the accuracy of GBLUP at predicting BLUP line mean variation in RPR, DTA, and EHT was assessed in the NCRPIS inbred diversity panel (Figure 5, Figure S4). This was accomplished with an IBS genomic relationship matrix constructed from 681,257 SNPs genotyped by GBS (Figure 5E). Even when 80% of the panel was used to calibrate a model to predict RPR variation in the remaining inbreds, only 38±4% of the BLUP variation in RPR was explained. This contrasted the 71% of variation explained by fitting the entire NCRPIS diversity panel (Figure 5F).

In a final assessment of genomic prediction accuracy, BLUE line means of RPR, DTA, and EHT between families and inbreds across environments were predicted by GBLUP (Figure S6). Correlation between BLUE and BLUP line means were weaker across RIL families before (RPR r  = 0.78, p<5e-3; DTA r  = 0.99, p<5e-3; EHT r  = 0.97, p<5e-3) than after accounting for between family variation (RPR r  = 0.83, p<5e-3; DTA r  = 0.99, p<5e-3; EHT r  = 0.99, p<5e-3). Higher GBLUP accuracies were obtained predicting BLUP than BLUE line means for RPR (Figure S6). Models calibrated from 20% of the inbreds stratified by family explained 21±2% of BLUE line mean variation for RPR. When fitting the total NAM panel, 52% of the variation was explained. Given their high heritability and minimal shrinkage of random genetic effects, comparable prediction accuracies for both BLUP and BLUE line mean estimators were attained for DTA, and EHT (Figure S6). BLUP and BLUE line means across the NCRPIS diversity panel were highly correlated (RPR r  = 0.87, p<5e-3; DTA r  = 0.99, p<5e-3; EHT r  = 0.99, p<5e-3) and wielded only slight reductions in prediction accuracy relative to BLUP line means (Figure S6).

## Discussion

In this study, we inferred the genetic architecture and genomic prediction accuracy of RPR as a proxy for maize stalk strength. In total, over 50,000 plants were measured for RPR across NAM and IBM families, and the NCRPIS diversity panel. These measures were collected in addition to DTA and EHT within three environments for NAM and IBM families, and two environments for the NCRPIS inbred diversity panel.

Transgressive segregation for RPR was observed in many NAM and IBM families (Figure 2). This finding contrasts past QTL analyses performed in families constructed from parents divergently selected for stalk strength and highlights the advantage of understanding the history of the germplasm under study [14]. Given alleles positively and negatively influencing RPR were not differentially pyramided and fixed by the divergent selection of progenitors in the NAM and IBM families, repulsion phase RPR QTL present in each inbred parent are a likely cause of increased variance in RPR among RILs relative to their parental difference. However, the shifted mean and asymmetry of RPR distributions among the RILs of many families relative to their mid-parent values suggest non-additive genetic variation may play a role in defining the genetic architecture of RPR. Given the inbred state of the NAM and IBM progenitors as well as their RIL progeny, this may be attributed to the presence of epistasis among loci. Results obtained for measures of both DTA and EHT across RIL families indicate these factors may play a role in the genetic architecture of these traits as well.

Despite the increased genetic variation in RPR among RILs relative to their parental values, estimates of broad sense heritability calculated on a line means basis were low in all families (Table 1). This contrasted DTA, EHT, and a previous RPR study performed in four biparental maize families distinct from this study [14]. Given the similarity in the RPR phenotyping method employed in both studies [14], this reduced heritability may be attributed to differing genetic architectures in the surveyed families. A reduction in the number or effect size of QTL segregating for RPR, or an increase in the linkage of opposing QTL for RPR may exist in several of the NAM and IBM families relative to the previous study. Multi-environment efforts were taken in both studies and likely mitigated the impact of environment on estimates of heritability. But, differences in the nature of the environments may also lead to divergence in estimates of variance explained by genetic-by-environment interactions. Our abilities to successfully model the potentially complex patterns of micro environmental variation in both studies may also differ and consequently influence estimates of error and thus heritability.

However, it is most likely the reduction in heritability resulted from the decreased replication of measures taken on each inbred in this study relative to the former multi-family RPR study [14]. The greatly increased number of RILs surveyed within this study, relative to previous studies [14], provided more effective recombination events, and increased mapping resolution. It also better ensured family-nested QTL were independent of confounding environmental variation and improved estimation of allele effects. But, the reduced replication of RPR measures collected on each inbred led to imprecision in estimation of line means and reduced heritability. The most powerful balance between replicating RPR measures on a given inbred and increasing the number of unique inbreds surveyed during mapping depends on the allele frequencies and linkage disequilibrium of QTL in the true model of genetic architecture. This true model is unknown and can only be approximated. As such, it may be instructive for future RPR mapping populations to be designed in a sequential manner [43], dynamically adapting population design to maximize power to best validate prior estimates of the true model of genetic architecture.

Of the heritable RPR variation that was observed in this study, approximately 60±4% was explained by variation between families. With a shared B73 founder, all SNPs differing between families also segregated within at least a fraction of the families depending on an allele’s frequency among founders. Variation between RIL families may then be attributed to mutation and ancestral recombination occurring between the non-B73 progenitor of each family; whereas, variation within families may be attributed largely to recent recombination. Differences in variance within and between families are likely due in part to the reduced functional diversity segregating within a single family relative to across families. But, given the random genetic effects used to model RPR, another reason for increased variance estimates between families, is an increased number of observations available to estimate between family variance. This increase in observations between families relative to within them reduced the error and the shrinkage of random effects modeling between family variance.

To resolve family-nested QTL explaining RPR variation, BLUP line means for RPR were used in bootstrapped joint-linkage mapping of the RIL families. After accounting for RPR variation between families, variation in families was mapped to family-nested molecular markers. This identified 78 family-nested markers with a RMIP over 10 (Table S3). Given the nature of bootstrapped joint-linkage mapping, two or more linked markers with an RMIP over 10 may explain RPR variation for the same family-nested QTL. For instance, a family-nested marker may be selected in model construction during one bootstrap sample; but, a linked family-nested marker capturing variation from the same family-nested QTL may be selected in another model.

While the identified number of family-nested QTL is highly dependent on the mapping resolution and method employed, the number entering into a single model for RPR in our approach was 18±2 before the permutation-based threshold for model entry was reached. Of these family-nested QTL, only about half were significant within a given NAM or IBM family. A single family-nested QTL model explained 81±3% of the variation across RPR BLUP line means. Supporting the presence of repulsion phase linkages of RPR loci in NAM parents, positive and negative allele effects relative to the common parent, B73, were observed within all families. The allele series present in all family-nested QTL may be attributed to the genetic background specificity of the effects and differing linkage patterns among nearby loci whose cumulative effect is explained by the family-nested QTL. Further highlighting the highly polygenic genetic architecture of RPR, all family-nested QTL effects were small and none explained over 2.7% of the BLUP variation in RPR across RILs after accounting for the between family variation. Although covariation was observed for DTA, EHT, and RPR among line and family means, and several loci for these traits co-localized, no significant mutually pleiotropic family-nested QTL were identified. This supports previous studies indicating ear height is a poor proxy for stalk strength during selection [14]. The modularity of the genetic architectures for these traits also suggests substantial flexibility exists in breeding to increase stalk strength in germplasm related to these RIL families.

After joint-linkage mapping, RPR variation between BLUP line means of the RIL families was further resolved by joint-linkage-assisted GWAS. This analysis revealed the segregation of 141 significant non-nested associations across families, after accounting for variation between families (Table S4). Further supporting estimates of modularity among the traits, no GWAS associations for RPR were shared with DTA or EHT associations mapped in joint-linkage-assisted GWAS. With the exception of the most robust associations, many RPR associations for GWAS did not co-localize with significant family-nested QTL. This may be a result of differing power in testing the family-nested QTL that allow for the positive and negative effects of an allele series, and that of the non-nested molecular markers queried for a unidirectional effect across all RIL families. Although co-localization of significant associations with previous studies was identified, the low resolution of past studies makes co-localization a probable event for all but the least complex traits [14].

In addition to joint-linkage-assisted GWAS, a sequential single marker linear mixed model GWAS of RPR BLUP line mean variation was performed in the NCRPIS diversity panel (Table S5). However, no highly significant associations with a false discovery rate below 10% were discovered. This paucity of significant associations in the NCRPIS diversity panel may be attributed to the low heritability of RPR and a lack of sufficient statistical power. Unlike the RIL families, where minor alleles are present in at least half the RILs of a single family and in most instances are found at much higher frequencies, rare alleles are responsible for a large proportion of the total genetic variance in the NCRPIS diversity panel. Also, the global population structure within this panel is substantial and each local subpopulation may have evolved differing genetic architectures for RPR leading to genetic background specificities and non-additive genetic variation that remain difficult to map across the entire panel.

Even in sizeable populations, such as the NCRPIS diversity panel, only the most common and largest additive effect alleles not severely confounded with population structure will significantly associate with a trait during a single marker mixed model GWAS. If the functional diversity of stalk strength is correlated with fitness and follows the Fisher-Orr model [44], many of the largest effect alleles are likely to be rare in the population as it nears optimal fitness. This suggests the large proportion of genetic diversity which persists as rare alleles in the NCRPIS diversity panel may contain the largest and most important alleles; however, we lack the power to find them. Also, if alleles for RPR are strongly confounded with population structure they will not be identified. Instead, their contributions to RPR variance will be explained by the IBS genomic relationship matrix. While it is necessary to account for population structure to overcome spurious associations during GWAS, this reduced false discovery rate may come at a steep cost to statistical power. Further studies in the NCRPIS diversity panel should seek to identify the importance of rare alleles by performing burden [45] or sequence kernel association tests [46]. Moreover, studies in populations that test these alleles approaching a frequency of 0.5 and nearer to linkage equilibrium are needed. This again suggests utility in the sequential design of mapping populations.

However, it remains to be determined if these studies should focus on candidate genes based on known molecular homology. GWAS associations identified as significant in the NAM and IBM families as well as the top 100 associations with a false discovery rate just over 10% in the NCRPIS diversity panel did not co-localize with genes involved in phenylpropanoid and cellulose synthesis, or vegetative phase transition. This lack of co-localization with genes in pathways known to influence many constituent traits of stalk strength may be due to their conservation in natural variation. This may result as a consequence of negative fitness effects. Nonetheless, given the low heritability of RPR, and high levels of diversity observed, it is likely the numerous anatomical and compositional factors influencing RPR greatly complicate mapping of causal loci. While little pleiotropy was observed between stalk strength and ear height or days to anthesis, interactions among other constituent traits may greatly influence stalk strength, and may do so in a non-additive manner. Future mapping efforts should decompose stalk strength into its constituent traits to reduce latent complexities limiting power and aid mapping of their potentially simpler genetic architectures.

Given the high degree of complexity in stalk strength genetic architecture inferred while mapping RPR, genomic prediction was performed using GBLUP to model relatedness based on an IBS relationship matrix. This method revealed substantial variation in BLUP and BLUE line means for RPR, DTA, and EHT was predictable using a fraction of RILs randomly selected from each NAM family (Figure 5, Figure S4, Figure S6). While BLUP and BLUE estimated line means for DTA and EHT produced nearly identical results due to their high heritabilities (Figure S4, Figure S6), BLUP line means for RPR (Figure 5) were significantly more predictable by GBLUP than BLUE line means (Figure S6). Both BLUP and BLUE line means for RPR were estimated in the first stage analysis without inclusion of a relatedness G structure for family or RIL nested within family. Interestingly, shrunken estimates of BLUP line means were better explained by the relationship matrix when fitted by GBLUP than BLUE line means for RPR. This may be a result of the similarity in biases imposed on RPR line mean estimates when terms for family and RIL nested within family are shrunken to their means as when GBLUP predicted line means are shrunken to the mean based on the relationship matrix. Future studies should perform a single stage analysis and include a genomic relationship matrix in estimation of BLUP line means if it is deemed significant by likelihood ratio testing.

As a comparison to GBLUP, the expected prediction accuracy of family-nested QTL models constructed by joint-linkage mapping subsets of the full data set was also assessed across the BLUP line mean variation for RPR. The variation explained in NAM by family-nested QTL models, 81% (Figure S5), and that explained by GBLUP, 80% (Figure 5) were comparable. But, upon cross-validation to assess prediction accuracy, family-nested QTL models possessed significantly lower accuracies than GBLUP within the RIL families at all surveyed calibration set sizes. The reduced accuracy of family-nested QTL relative to GBLUP may result not only from the modeling approach taken; but also from the unique marker data available. In the family-nested QTL model, QTL were selected from a set of 1,106 markers; but, the model was also provided information detailing to which family each RIL belonged and QTL were nested within these families. In GBLUP, the 1.6 million markers of the maize HapMapV1 [18] were provided. Further testing of cross-validated models constructed in joint-linkage-assisted GWAS of the 1.6 million markers in the maize HapMap is needed. This will help to evaluate the relative merits of marker effect selection and shrinkage approaches on the expected prediction accuracies of RPR in the NAM panel.

In the NCRPIS diversity panel, GBLUP was also performed to assess expected prediction accuracies. This was accomplished with an IBS relationship matrix estimated from 681,257 SNPs. While significant prediction accuracies were attained, they remained lower than those observed in the NAM panel. The increased genetic diversity and reduced marker density in the NCRPIS panel relative to the NAM and IBM families may be attributed to this lower prediction accuracy. The potential complexity of the underlying genetic architecture for each trait increases as the diversity in the population increases, the marker density and size of the calibration set limit the degree that GBLUP is both informed of, and able to explain this complexity. Also, given the highly structured nature of the NCRPIS panel and the prevalence of rare alleles, the genetic architecture explaining variation in the surveyed traits may have evolved by differing mechanisms within each subpopulation of the diversity panel. This may introduce genetic background specificity and non-additivity into modeling the global population and is a potential reason for the more limited prediction accuracy of GBLUP in the NCRPIS diversity panel relative to the NAM panel.

While the expected RPR prediction accuracy of a given calibration set size is informative, variation in prediction accuracies was present at all the calibration set sizes in both NAM and NCRPIS diversity panels. Variations in accuracy were especially high within the family-nested QTL models in the NAM panel. This suggests the exact set of lines chosen to calibrate a model may have a larger influence on prediction accuracy when family-nested QTL or other marker effects are selected than when a GBLUP model is assumed and all effects are included but shrunken to the mean. In GBLUP, RPR prediction accuracies did not significantly differ if lines included in a calibration set were randomly selected across families, or were stratified by family. Given the large fraction of BLUP line mean variation in RPR explained by between family variation, family stratified sampling should ensure this variation is better modeled. However, a randomly selected 20% of the NAM panel appeared to sufficiently sample most families to accurately capture between family RPR variation. Previous studies have revealed the gains to be had from strategically sampling the phenotypic/genotypic space when developing calibration sets for genomic prediction [47]–[49], and algorithms have been developed to select improved calibration sets [50]. Given only five sizes of calibration sets were screened for random and family-stratified RPR prediction accuracy, further testing of calibration sets, especially those smaller than 20% of the NAM panel, is needed to determine those properties enhancing reliability of stalk strength prediction by GBLUP and other genomic prediction methods.

Stalk strength and its constituent traits will continue to play an important role in the selection of maize varieties maximizing grain harvestability and silage desirability. This study presents a comprehensive look at the genetic architecture of stalk strength and an assessment of its prediction accuracy. Future efforts detailing the genetic architecture of the anatomical, compositional, and phase change traits underlying stalk strength may reveal simpler architectures and better leverage our existing biological knowledge to understand the genetics of stalk strength. Understanding the genetic architecture of these traits and employing crop modeling to realize how they interact to influence stalk strength is a logical path to better inform plant breeding decisions. Breeding efforts in low heritability traits like stalk strength must incorporate a present, but inherently limited, understanding of genetic architecture and an ability to predict breeding values into a unified selection framework. These efforts will improve prediction of a population’s response to frequency-dependent selection on alleles, haplotypes, and genotypes. Given the availability of inexpensive sequence data, this is increasingly becoming an achievable goal in the optimization of crop improvement.

## Supporting Information

Figure S1 **DTA and EHT transgressive segregation within the RIL families and NCRPIS diversity panel.**
(TIF)

Figure S2 **RPR, DTA, and EHT correlations within the RIL families and NCRPIS diversity panel.**
(TIF)

Figure S3 **RPR, DTA, and EHT associations within and across the RIL families and NCRPIS diversity panel.**
(TIF)

Figure S4 **GBLUP of DTA and EHT BLUP line means across the RIL families and NCRPIS diversity panel.**
(TIF)

Figure S5 **Family-nested QTL-based prediction of RPR BLUP line means across the RIL families.**
(TIF)

Figure S6 **GBLUP of RPR, DTA, and EHT BLUE line means across the RIL families and NCRPIS diversity panel.**
(TIF)

Table S1 RPR, DTA, and EHT BLUP and BLUE line means across the RIL families.
(TXT)

Table S2 RPR, DTA, and EHT BLUP and BLUE line means across the NCRPIS diversity panel.
(TXT)

Table S3 RPR, DTA, and EHT family-nested QTL associations within the RIL families.
(TXT)

Table S4 RPR joint-linkage-assisted GWAS results across the RIL families.
(TXT)

Table S5 RPR linear mixed model GWAS results across the NCRPIS diversity panel.
(TXT)
