# Supplementary figures and images for: The Genetic Architecture of Maize Stalk Strength
Source: PLoS One. 2013 Jun 20;8(6):e67066. doi: 10.1371/journal.pone.0067066 (PMC3688621; doi:10.1371/journal.pone.0067066)

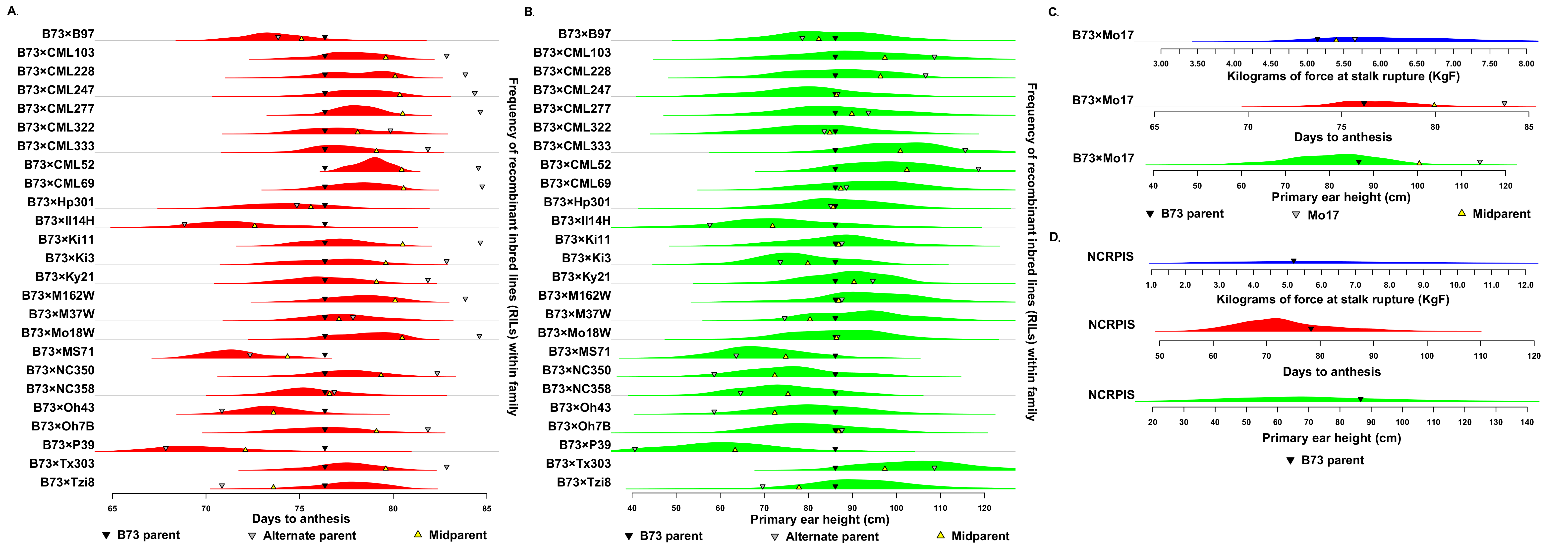

Supplement: Figure S1 — DTA and EHT transgressive segregation within the RIL families and NCRPIS diversity panel. (TIF) [file pone.0067066.s001.tif]

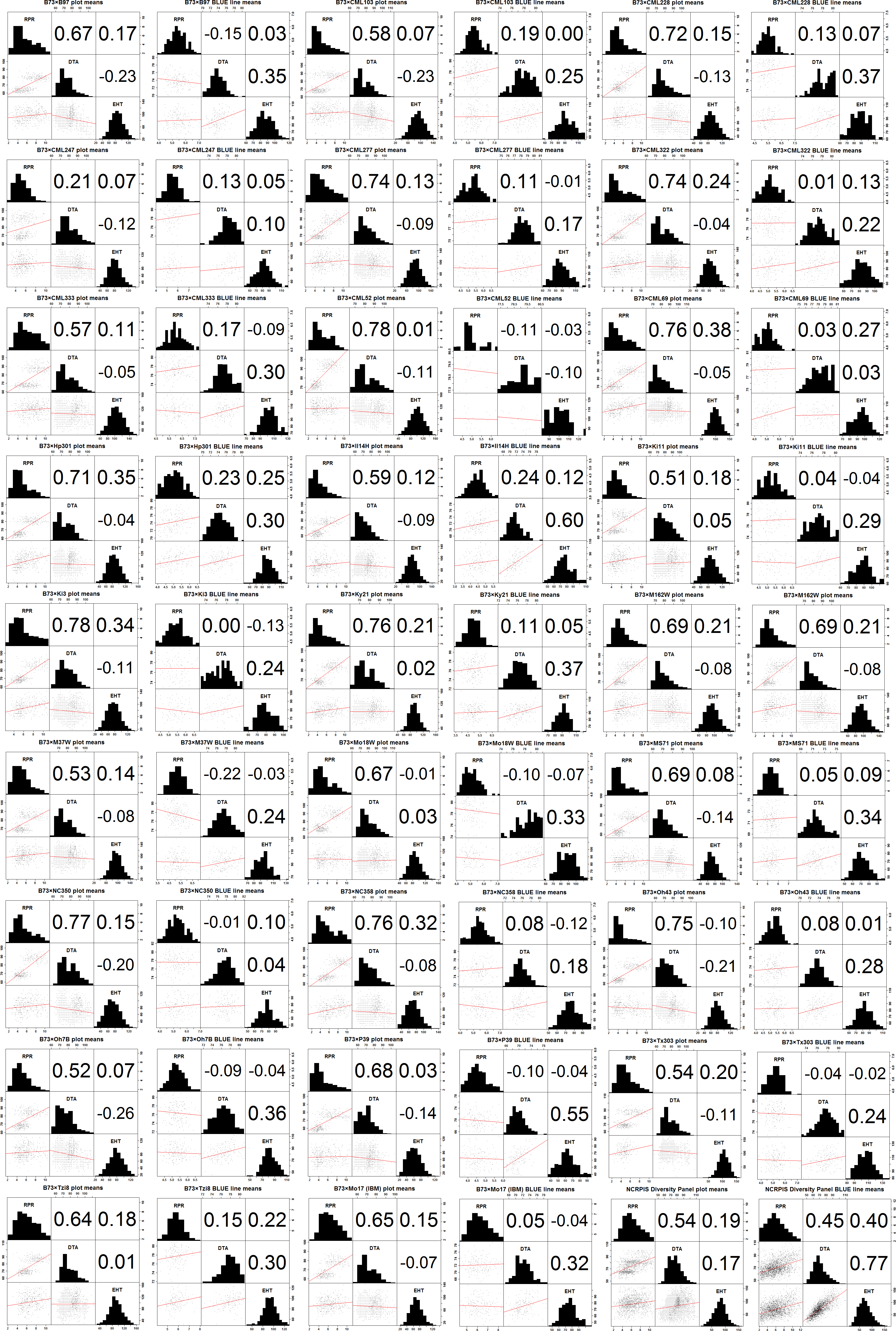

Supplement: Figure S2 — RPR, DTA, and EHT correlations within the RIL families and NCRPIS diversity panel. (TIF) [file pone.0067066.s002.tif]

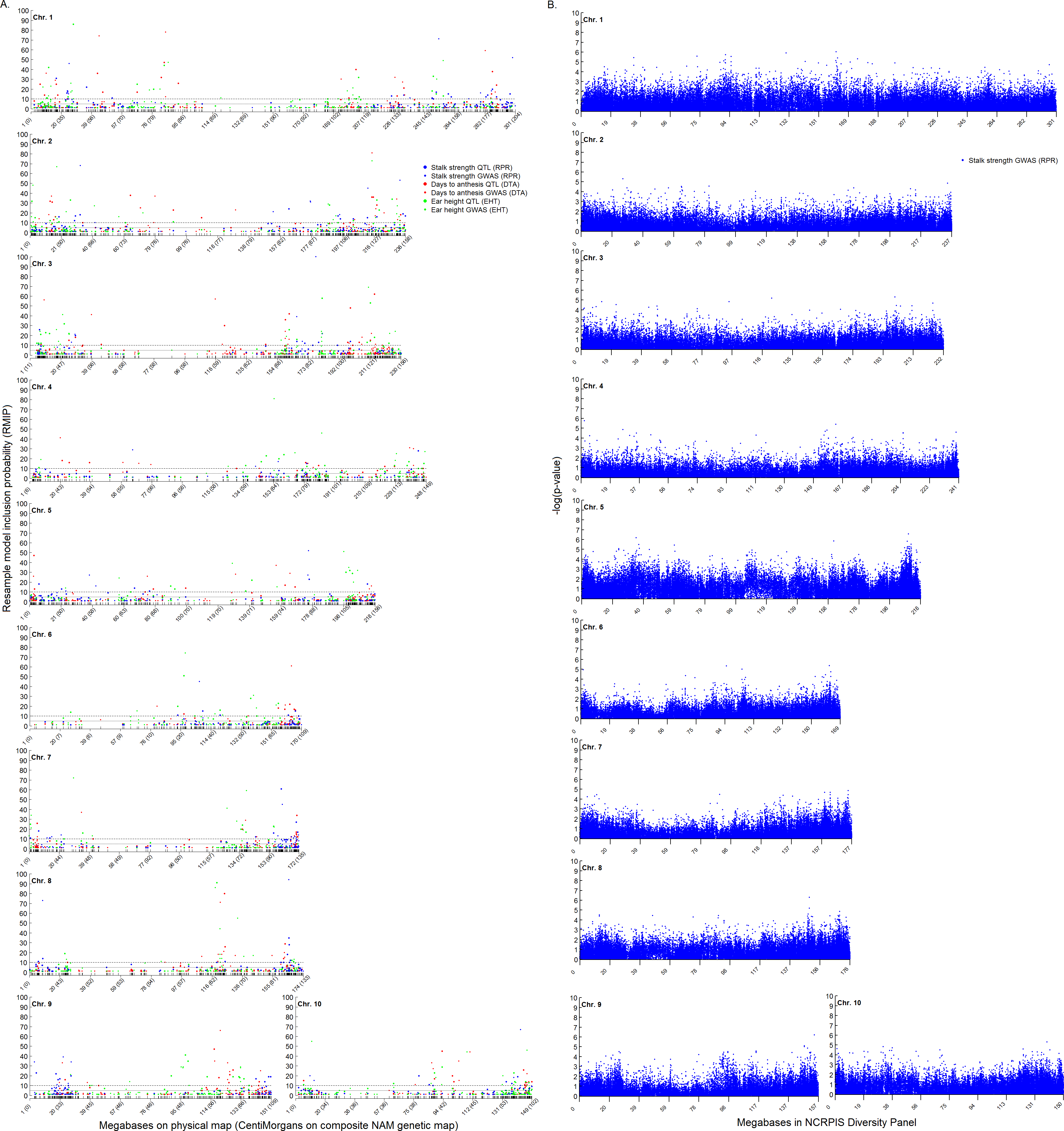

Supplement: Figure S3 — RPR, DTA, and EHT associations within and across the RIL families and NCRPIS diversity panel. (TIF) [file pone.0067066.s003.tif]

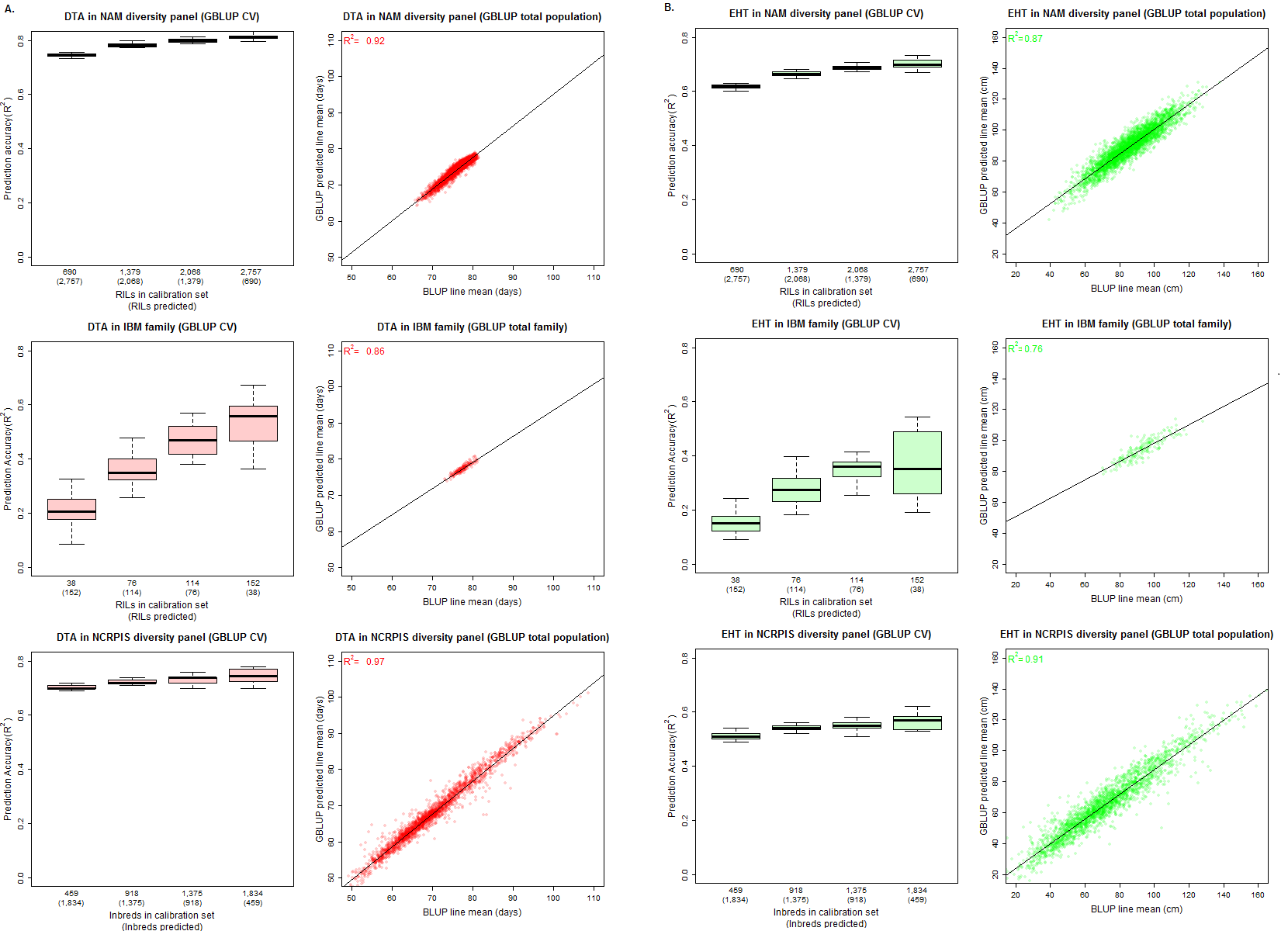

Supplement: Figure S4 — GBLUP of DTA and EHT BLUP line means across the RIL families and NCRPIS diversity panel. (TIF) [file pone.0067066.s004.tif]

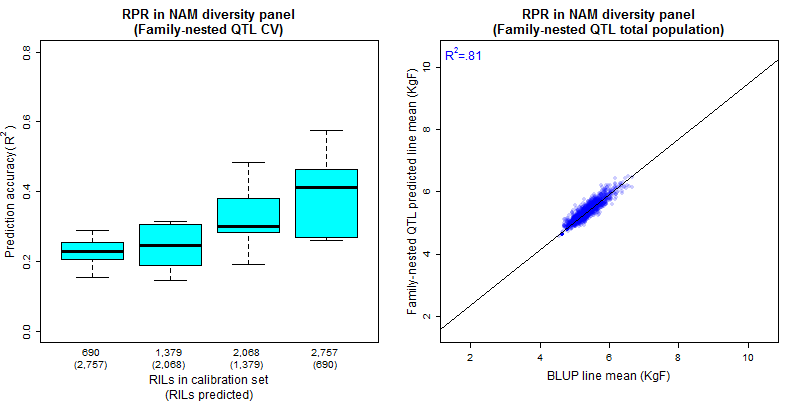

Supplement: Figure S5 — Family-nested QTL-based prediction of RPR BLUP line means across the RIL families. (TIF) [file pone.0067066.s005.tif]

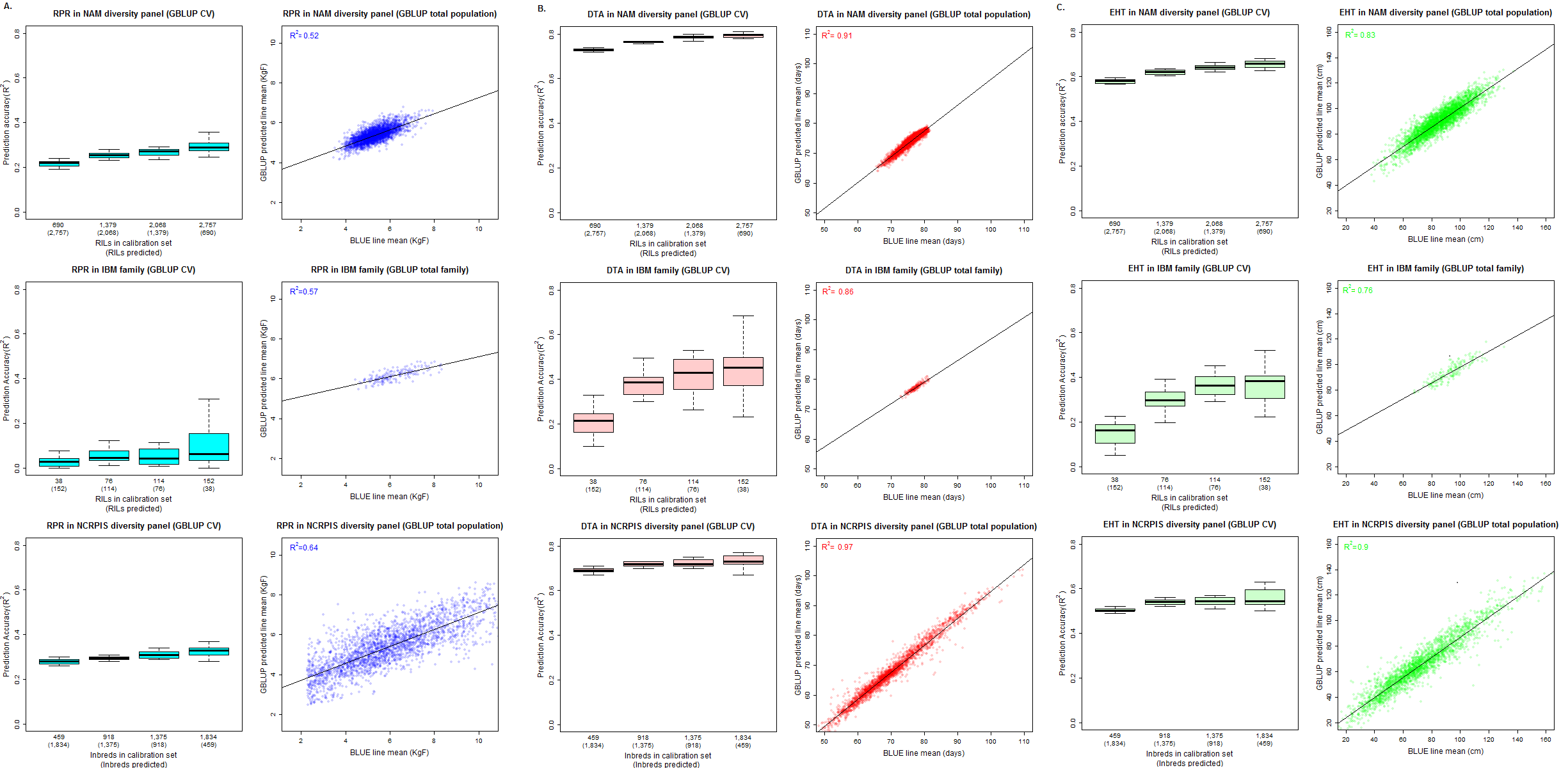

Supplement: Figure S6 — GBLUP of RPR, DTA, and EHT BLUE line means across the RIL families and NCRPIS diversity panel. (TIF) [file pone.0067066.s006.tif]
